# Efficacy and safety of nintedanib in advanced idiopathic pulmonary fibrosis

**DOI:** 10.1186/s12931-018-0907-8

**Published:** 2018-10-19

**Authors:** Hee-Young Yoon, Sojung Park, Dong Soon Kim, Jin Woo Song

**Affiliations:** Department of Pulmonary and Critical Care Medicine, Asan Medical Centre, University of Ulsan College of Medicine, Olympic-Ro 43-Gil, Songpa-Gu, Seoul, 05505 Republic of Korea

**Keywords:** Idiopathic pulmonary fibrosis, Respiratory function tests, Nintedanib, Treatment outcome, Adverse effects

## Abstract

**Background:**

Phase 3 trials have shown that nintedanib reduces the decline in forced vital capacity (FVC) in patients with mild-to-moderate idiopathic pulmonary fibrosis (IPF) with acceptable safety profiles; however, its effects on advanced IPF are unclear. We investigated the efficacy and safety of nintedanib in patients with advanced IPF.

**Methods:**

Prospective data were obtained from 108 IPF patients administered at least one dose of nintedanib. Of these patients, 47.2% had advanced IPF (FVC < 50% predicted, or diffusing capacity < 30% predicted).

**Results:**

The median treatment duration was 42.2 weeks. Nintedanib significantly reduced the decline rate in both FVC (− 0.55% [before] vs. -0.32% [after] predicted/month, *p* = 0.020) and total lung capacity (TLC) (− 0.35% vs. -0.06% predicted/month, *p* < 0.001) in all patients. A significant improvement in FVC decline rate after treatment was also observed in the advanced group (− 0.77% vs. -0.22% predicted/month, *p* = 0.003), but not in the non-advanced group (− 0.41% vs. -0.33% predicted/month, *p* = 0.564). Adverse events occurred in 97.2% of the cohort, including diarrhoea (50.0%) and anorexia (45.4%). Following adjustment for treatment duration, no inter-group difference in odds ratio was observed for the occurrence of adverse events. However, the advanced group showed a higher frequency of treatment interruption (68.0% vs. 40.0%), mainly as a result of disease progression (47.1% vs. 36.4%).

**Conclusions:**

The efficacy and safety profiles of nintedanib in the advanced group were comparable to those in the non-advanced group except for a higher frequency of discontinuation, which may be due to the advanced status itself.

**Electronic supplementary material:**

The online version of this article (10.1186/s12931-018-0907-8) contains supplementary material, which is available to authorized users.

## Background

Idiopathic pulmonary fibrosis (IPF) is a chronic, progressive, fibrosing interstitial pneumonia characterized by poor prognosis [1, 2]. The pathogenesis of IPF is suggested to involve several profibrotic mediators, such as fibroblast growth factors (FGFs), platelet-derived growth factors (PDGFs), and transforming growth factor beta [3, 4]. Nintedanib, an intracellular tyrosine kinase inhibitor that targets vascular endothelial growth factor, FGFs, and PDGF receptors, has shown anti-fibrotic and anti-inflammatory activity in a preclinical study [5, 6]. Recent randomised controlled trials of patients with mild-to-moderate IPF revealed that nintedanib reduced the rate of decline of forced vital capacity (FVC) and disease progression, and that adverse events (AEs) were well tolerated [7, 8].

Limited data have suggested that nintedanib may also stabilise the decline in lung function in patients with advanced IPF [9]. Post-hoc subgroup analyses of pooled data from two phase 3 trials, in which patients with IPF were classified according to baseline FVC % predicted (≤ 70%, > 70%; ≤ 80%, > 80%; or ≤ 90%, > 90%), showed no statistically significant inter-group differences in the effect of nintedanib on the annual rate of decline in FVC [10–12]. In an exploratory subgroup analysis of the open-label extension trial, INPULSIS-ON, in which patients were classified according to baseline FVC % predicted (≤ 50%, > 50%), the decline in FVC in both subgroups was similar to that observed in the phase 3 trials. However, the number of patients with FVC ≤ 50% predicted was small (*n* = 24), and the efficacy and safety analyses were only descriptive [9].

The aim of the present study was to compare the efficacy and safety of nintedanib in patients with advanced and non-advanced IPF.

## Methods

### Study subjects

**Fig. 1:**
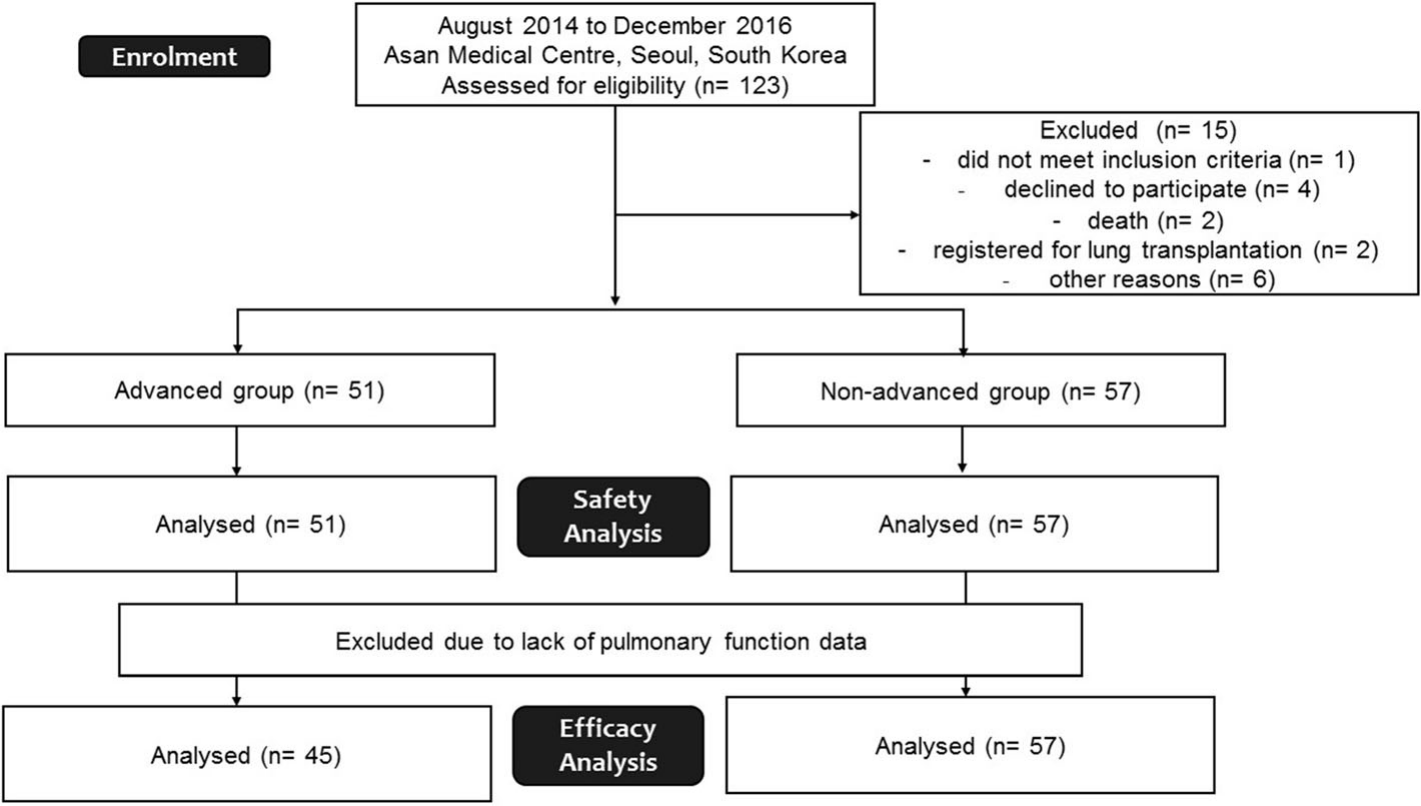
Enrolment of the study subjects

Between August 2014 and December 2016, a total of 108 patients with IPF who received nintedanib through the named patient use programme at Asan Medical Centre, Seoul, Republic of Korea, were recruited for this study (Fig. 1). The inclusion and exclusion criteria of the named patient use programme are shown in Additional file 1: Table S1. IPF was confirmed in 42.7% of the 108 patients via biopsy (37.4% in the advanced group and 45.6% in the non-advanced group). All patients fulfilled the IPF diagnostic criteria of the American Thoracic Society (ATS)/European Respiratory Society/Japanese Respiratory Society/Latin American Thoracic Association [1]. Patients with baseline FVC < 50% predicted, or diffusing capacity for carbon monoxide (DLco) < 30% predicted, were classified as advanced IPF cases. The study was approved by the Institutional Review Board of the Asan Medical Centre (2014–0805) and all patients provided written informed consent prior to inclusion.

### Clinical variables

The data on baseline demographics, lung function tests, and the 6 min walk test (6MWT) were obtained at the time of nintedanib initiation. Spirometry to measure FVC, plethysmography to measure total lung capacity (TLC) and evaluation of DLco were performed at 3 month intervals, in accordance with the recommendations of ERS/ATS [13–15]. The results were expressed as percentages of normal predicted values. The 6MWT was performed in accordance with ATS guidelines at the time of initiation of nintedanib with the following minor modification: the technician continually monitored the oxygen saturation levels of the patient with no additional remark or encouragement [16].

### Evaluation of efficacy

Efficacy was evaluated in all patients who were treated with at least one dose of nintedanib and underwent pulmonary function tests at least three times in each pre- and post-treatment period. The decline rate of lung function, which was estimated by the mixed-effects model, was compared between before and after treatment, or between the advanced and non-advanced group. The adjusted lung function values at each time point in the pre- and post-treatment periods were compared with baseline values. For categorical comparisons, disease progression was defined as a ≥ 10% absolute decline in FVC over 12 months (FVC_after 12 months_ – FVC_baseline_).

### Evaluation of safety

Safety was assessed in all patients who received at least one dose of nintedanib. AEs were monitored from the initiation of treatment to 28 days after the final dose of nintedanib and assessed at each follow-up visit by physician’s examination. The time of occurrence and termination was recorded. The AE terms were classified in accordance with the *Medical Dictionary for Regulatory Affairs*. Acute exacerbation was defined as an acute, clinically significant deterioration of unidentifiable cause in a patient with IPF [17]. Serious AEs (SAEs) were defined as adverse drug events occurring at any dose that resulted in one of the following outcomes: 1) death; 2) life-threatening hospitalization; 3) disability or permanent damage; 4) any event that required intervention to prevent permanent impairment or damage; or 5) any other serious medical event.

### Statistical analysis

Continuous variables are expressed as the mean ± standard deviation (SD) or median with interquartile range (IQR) and the categorical variables are expressed as percentages. Continuous variables were analysed by using Student’s *t*-test or the Mann-Whitney *U* test. Categorical variables were analysed by using Pearson chi-square test or the Fisher exact test. A mixed-effects model was used to examine the rate of decline of lung function and the adjusted mean changes from baseline, using age, sex, body mass index (BMI), and smoking status as fixed effects, and time-by-person (slope) as a random effect. To compare the efficacy between the advanced and non-advanced groups, or between the pre- and post-treatment periods, the time-by-group or time-by-treatment period was used as fixed effects in addition to the mixed-effects model. The odds ratio (OR) and 95% confidence interval (CI) for the occurrence of AEs in the advanced group compared with that in the non-advanced group were adjusted for treatment duration by using binary logistics regression analysis. All tests for significance were two-sided, and variables with *p*-values of < 0.05 were considered to indicate statistical significance. All statistical analyses were computed by using SPSS version 23.0 (SPSS Inc., Chicago, IL, USA).

## Results

### Baseline characteristics of the cohort

The median follow-up duration was 43.7 weeks (IQR: 22.9–70.0 weeks). The mean age of patients was 66.7 years; 79.6% of the patients were men and 47.2% were classified into the advanced group (Table 1). The median duration of exposure to nintedanib was 42.2 weeks (IQR: 18.9–66.8 weeks; 26.7 weeks in the advanced group and 57.0 weeks in the non-advanced group, *p* < 0.001). Compared with the patients in the non-advanced group, those in the advanced group were less likely to be current smokers, and tended to have a lower BMI, poorer lung function and exercise capacity, higher mean estimated systolic pulmonary arterial pressure, and more frequent use of steroids (Table 1).

**Table 1:** Comparison of the baseline characteristics between the advanced and non-advanced idiopathic pulmonary fibrosis groups

Characteristic	Total	Advanced	Non-advanced	*P*-value
Number of patients	108	51	57	
Age, years	66.7 ± 7.9	66.4 ± 8.6	68.2 ± 7.2	0.236
Male	86 (79.6)	39 (76.5)	47 (82.5)	0.441
Smoking status				0.027
Never smoker	25 (23.1)	16 (31.4)	9 (15.8)	
Former smoker	79 (73.1)	35 (68.6)	44 (77.2)	
Current smoker	4 (3.7)	0 (0.0)	4 (7.0)	
Body mass index, kg/m^2^	23.3 ± 3.2	22.7 ± 3.4	24.2 ± 2.7	0.018
Time since diagnosis, months	39.7 ± 32.0	41.2 ± 35.3	38.9 ± 29.1	0.709
Comorbidities				
Diabetes mellitus	22 (20.4)	9 (17.6)	13 (22.8)	0.506
Cardiovascular disease	11 (10.2)	4 (7.8)	7 (12.3)	0.447
Cerebrovascular disease	4 (3.7)	3 (5.9)	1 (1.8)	0.642
Chronic liver disease	3 (2.8)	2 (3.9)	1 (1.8)	0.601
Pulmonary function test				
FVC, % predicted	56.2 ± 15.1	46.8 ± 9.5	65.3 ± 13.5	< 0.001
DLco, % predicted	35.7 ± 12.6	27.1 ± 9.8	43.6 ± 9.4	< 0.001
TLC, % predicted	58.0 ± 12.2	50.8 ± 7.6	65.0 ± 11.5	< 0.001
6MWT				
Distance, m	341.2 ± 122.5	275.0 ± 107.4	411.9 ± 96.4	< 0.001
Lowest SaO_2_, %	84.2 ± 5.3	82.8 ± 4.8	87.4 ± 4.8	< 0.001
SPAP, mmHg^a^	36.4 ± 1.1	38.8 ± 13.0	34.2 ± 9.2	0.039
Previous treatment				
Pirfenidone	20 (18.5)	10 (19.3)	10 (17.5)	0.783
Prednisolone	17 (15.7)	13 (25.5)	4 (7.0)	0.008
Immunosuppressants	11 (10.2)	9 (17.6)	2 (3.5)	0.015
*N*-Acetylcysteine	5 (4.6)	0 (0.0)	5 (8.8)	0.059
None	66 (60.6)	26 (51.0)	40 (70.2)	0.041

Data are presented as mean ± standard deviation or number (%), unless otherwise indicated

*FVC* Forced vital capacity, *DLco* Diffusing capacity of the lung for carbon monoxide, *TLC* Total lung capacity, *6MWT* 6 min walk test, *SaO*_*2*_ Saturation of oxygen, *SPAP* Estimated systolic pulmonary arterial pressure

^a^Measured by echocardiography

### Lung function decline rates

The efficacy was evaluated in 102 patients (45 in the advanced IPF group and 57 in the non-advanced IPF group) and the median treatment duration was 44.1 weeks (29.1 weeks in the advanced group and 57.0 weeks in the non-advanced group, *p* < 0.001).

**Fig. 2:**
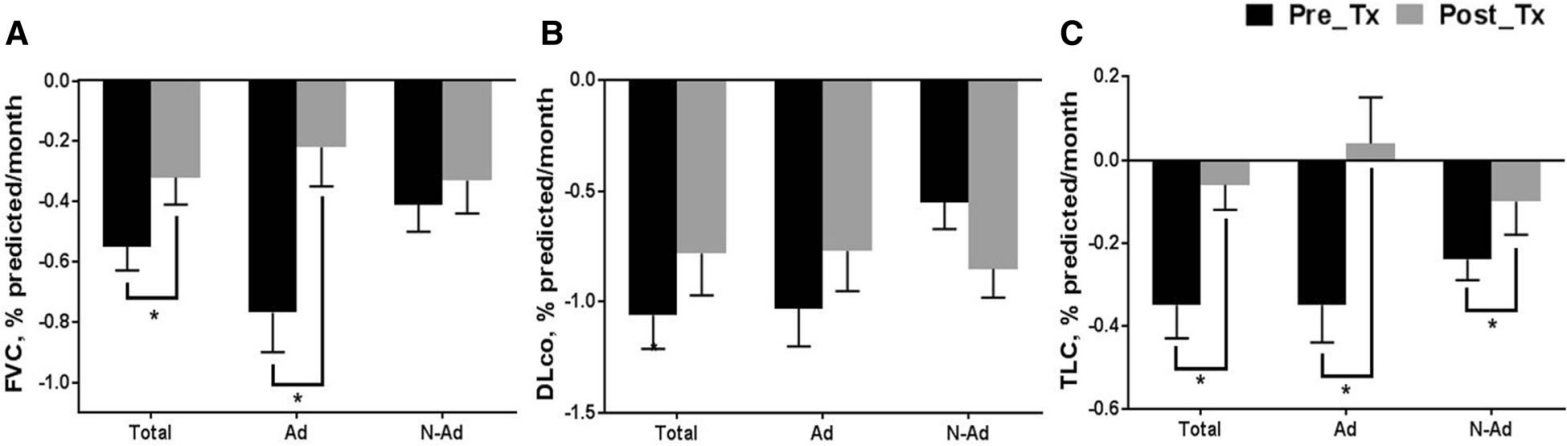
Rate of decline in lung function before and after treatment. **a** The rate of decline in FVC before and after treatment. **b** The rate of decline in DLco before and after treatment. **c** The rate of decline in TLC before and after treatment. Each bar and line represent the mean and standard deviation. *p < 0.05. Total, total patients; Ad, advanced group; N-Ad, non-advanced group; Pre_Tx, before treatment; Post_Tx, after treatment; FVC, forced vital capacity; DLco, diffusing capacity of the lung for carbon monoxide; TLC, total lung capacity

In all patients, the decline rate in FVC was significantly reduced after treatment (− 0.55% ± 0.08% [before] vs. -0.32% ± 0.09% [after] predicted/month, *p* = 0.020; Fig. 2). Similar trends were observed for TLC. In contrast, the decline rate in DLco was not changed after treatment (− 1.60 ± 0.15% vs. -0.78 ± 0.19% predicted/month, *p* = 0.921).

In the advanced IPF group, the decline rate in FVC decreased after treatment (− 0.77% ± 0.13% [before] vs. -0.22% ± 0.13% [after] predicted/month, *p* = 0.003; Fig. 2). However, only a numerical improvement in FVC decline rate after treatment was observed in the non-advanced group (− 0.41% ± 0.09% vs. -0.33% ± 0.11% predicted/month, *p* = 0.564). There was a significant difference in FVC decline rate between the advanced and non-advanced groups before treatment (− 0.77% ± 0.13% [advanced] vs. -0.41% ± 0.09% [non-advanced] predicted/month, *p* = 0.018), but not after treatment (− 0.22% ± 0.13% vs. -0.33% ± 0.11% predicted/month, p = 0.564). In both groups, the decline rate in TLC was significantly reduced after treatment, which was not the case for DLco.

### Lung function changes

**Fig. 3:**
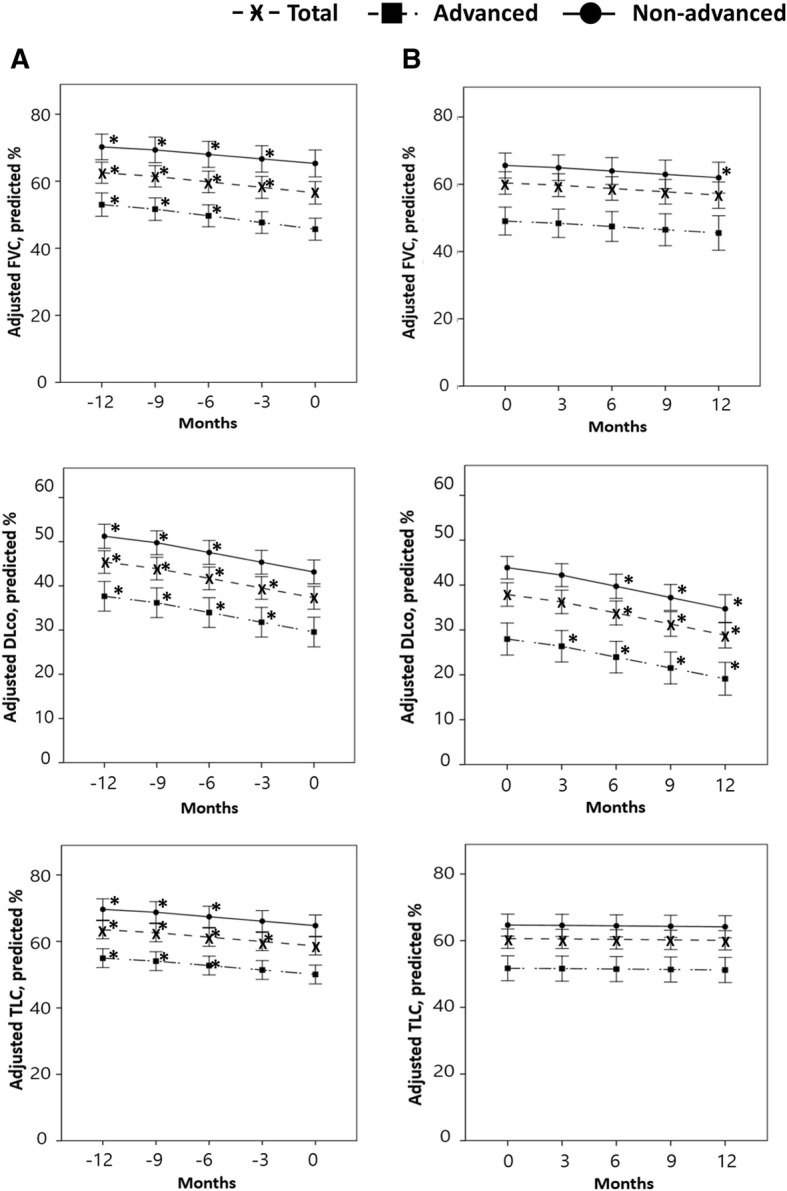
Adjusted changes in lung function before and after treatment. **a** Serial adjusted changes in FVC, DLco, and TLC before treatment. **b** Serial adjusted changes in FVC, DLco, and TLC after treatment. Each dot and error bar represent the mean and 95% confidence interval. *p < 0.05 (compared with baseline values). Total, total patients; FVC, forced vital capacity; DLco, diffusing capacity of the lung for carbon monoxide; TLC, total lung capacity

The adjusted mean FVC changes from baseline were significant before treatment (ΔFVC: − 6.4% ± 0.9% predicted, *p* < 0.001 [12 months]; − 3.0% ± 0.6% predicted, *p* < 0.001 [6 months]), but were not significant after treatment (ΔFVC: − 1.4% ± 1.2% predicted, *p* = 0.227 [6 months]; − 3.0% ± 1.5% predicted, *p* = 0.055 [12 months]; Fig. 3). The adjusted differences in TLC showed a trend similar to that of FVC; however, the differences in DLco from baseline were significant before and after treatment.

In the advanced group, the adjusted mean FVC changes from baseline were significant before treatment (ΔFVC: − 9.5% ± 1.3% predicted, *p* < 0.001 [12 months]; − 3.4% ± 0.9% predicted, *p* < 0.001 [6 months]), but not after treatment (ΔFVC: − 1.7% ± 1.3% predicted, *p* = 1.000 [6 months]; − 1.4% ± 1.5% predicted, *p* = 0.403 [12 months]; Fig. 3). The results for the non-advanced group were comparable; however, the changes in FVC at 12 months after treatment were significant (− 3.5% ± 1.7% predicted, *p* = 0.043). The adjusted differences in TLC showed a trend similar to that of FVC in both groups; however, the differences in DLco from baseline were significant before and after treatment.

### Disease progression

**Fig. 4:**
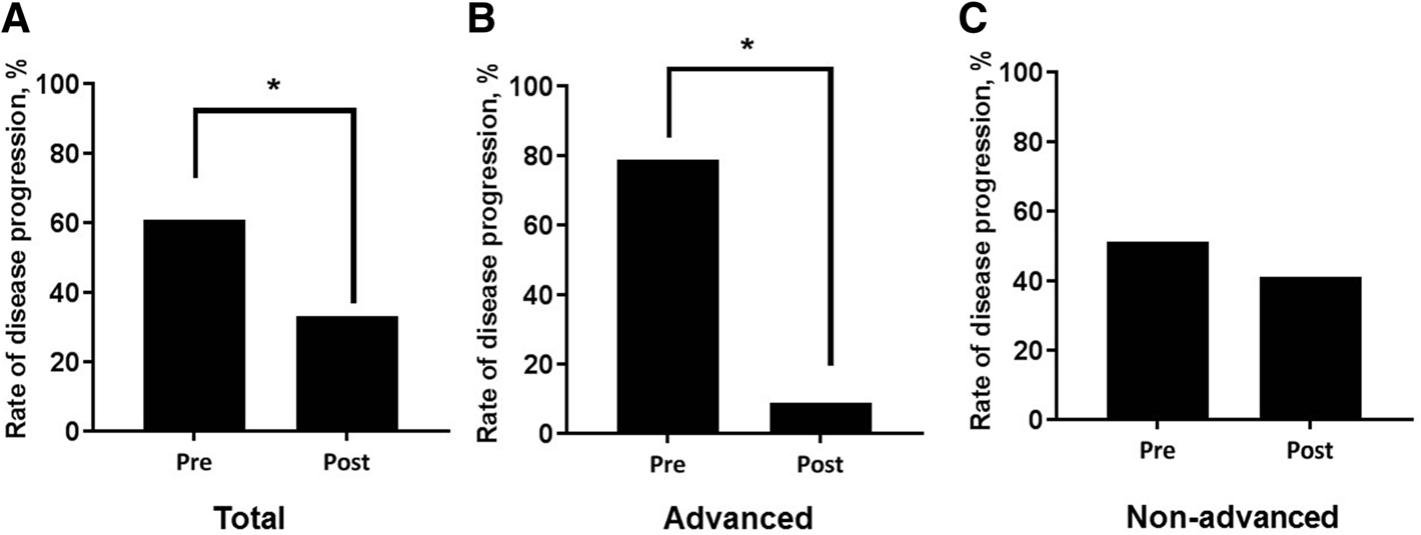
Categorical changes in lung function before and after treatment. **a** Total cohort; **b** Advanced group; **c** Non-advanced group. Disease progression was defined as a ≥ 10% absolute decline in FVC over 12 months. Pre, pre-treatment; Post, post-treatment

In the total cohort, a significant reduction in the rate of disease progression was observed after treatment (61.1% [before] vs. 33.3% [after], *p* = 0.008; Fig. 4). Similar trends were observed in the advanced group (78.9% vs. 9.1%, *p* < 0.001); however, a numerical improvement after treatment was only observed in the non-advanced group (51.4% vs. 41.2%, *p* = 0.472). Compared with the non-advanced group, the rate of disease progression in the advanced group was significantly higher (78.9% [advanced] vs. 51.4% [non-advanced], *p* = 0.048) before treatment and lower (9.1% vs. 41.2%, *p* = 0.050) after treatment.

### Adverse events

Of the 108 patients who received at least one dose of nintedanib, 97.2% experienced AEs (Table 2). The most frequent AE was diarrhoea (50.0%), followed by anorexia (45.4%), nausea/vomiting (24.1%), dyspepsia (18.5%) and cough (18.5%). No difference in the incidence of AEs was observed between the advanced and non-advanced groups (98.0% vs. 95.5%, *p* = 1.000). The advanced group had a lower frequency of diarrhea (37.3% vs. 61.4%, *p* = 0.012) and a higher frequency of nausea/vomiting (33.3% vs. 15.8%, *p* = 0.033) than the non-advanced group; however, no inter-group differences were found in terms of the OR in the occurrence of AEs after adjustment for treatment duration (Additional file 1: Table S2). The median time-of-onset for AEs was 4.3 weeks (IQR: 1.6–11.7 weeks), and no inter-group difference was observed between the advanced and non-advanced groups (3.9 vs. 4.9 weeks, *p* = 0.373), except for anorexia (5.0 weeks vs. 17.6 weeks, *p* = 0.042; Additional file 1: Table S3).

**Table 2:** Comparison of adverse events between the advanced and non-advanced idiopathic pulmonary fibrosis groups

Characteristic	Total	Advanced	Non-advanced	*P*-value
Number of patients	108	51	57	
Adverse events	105 (97.2)	50 (98.0)	55 (95.5)	1.000
Diarrhoea	54 (50.0)	19 (37.3)	35 (61.4)	0.012
< 4 times/day	38 (35.2)	15 (29.4)	23 (4.0)	0.264
4–6 times/day	13 (12.0)	2 (3.9)	11 (19.3)	0.014
> 6 times/day	3 (2.8)	2 (3.9)	1 (1.8)	0.601
Anorexia	49 (45.4)	27 (52.9)	22 (38.6)	0.135
Nausea/vomiting	26 (24.1)	17 (33.3)	9 (15.8)	0.033
Dyspepsia	20 (18.5)	8 (15.7)	12 (21.1)	0.541
Cough	20 (18.5)	9 (17.6)	11 (19.3)	0.825
Dyspnoea	16 (14.8)	10 (19.6)	6 (10.5)	0.185
Weight loss	16 (14.8)	8 (15.7)	8 (14.0)	0.809
General weakness	14 (13.0)	9 (17.6)	5 (8.8)	0.170
Upper respiratory infection	11 (10.2)	4 (7.8)	7 (12.3)	0.447
Hepatotoxicity				
> 3× ULN	9 (8.3)	6 (11.8)	3 (5.3)	0.302
> 5× ULN	4 (3.7)	3 (5.9)	1 (1.8)	0.342

Data are presented as numbers (%), unless otherwise indicated

*ULN* Upper limit of normal

### Serious adverse events

SAEs were reported in 54.6% of patients in the cohort. The most common SAE was pneumonia (27.8%; Table 3). Cardiac disorders occurred in three patients (2.8%): acute myocardial infarction, stable angina, and atrial fibrillation. No inter-group differences in the incidence of SAEs (62.7% [advanced] vs. 47.4% [non-advanced], *p* = 0.109) and the adjusted OR for SAEs were observed. However, the patients in the advanced subgroup had a tendency for a higher risk of pneumothorax (OR: 8.611, 95% CI: 0.851–87.135, *p* = 0.068; Additional file 1: Table S4).

**Table 3:** Comparison of serious adverse events between the advanced and non-advanced idiopathic pulmonary fibrosis groups

Characteristic	Total	Advanced	Non-advanced	*P*-value
Number of patients	108	51	57	
Serious adverse events	59 (54.6)	32 (62.7)	27 (47.4)	0.109
Pneumonia	30 (27.8)	16 (31.4)	14 (24.6)	0.430
Acute exacerbation	9 (8.3)	5 (9.8)	4 (7.0)	0.732
Pneumothorax	6 (5.6)	5 (9.8)	1 (1.8)	0.098
Lung cancer	4 (3.7)	1 (2.0)	3 (5.3)	0.620
Pulmonary thromboembolism	3 (2.8)	1 (2.0)	2 (3.5)	1.000
Cardiac disorders^a^	3 (2.8)	1 (2.0)	2 (3.5)	1.000
Cerebral vascular disease	1 (0.9)	1 (2.0)	0 (0.0)	0.472
Death	18 (16.7)	11 (21.6)	7 (12.3)	0.196
Pneumonia	10 (9.3)	7 (13.7)	3 (5.3)	0.186
Acute exacerbation	2 (1.9)	0 (0.0)	2 (1.9)	0.497
Severe liver injury^b^	1 (0.9)	0 (0.0)	1 (1.8)	1.000
Cancer progression	1 (0.9)	1 (2.0)	0 (0.0)	0.472
Myocardial infarction	1 (0.9)	0 (0.0)	1 (1.8)	1.000
Unknown	3 (2.8)	1 (2.0)	2 (3.5)	1.000

Data are presented as numbers (%), unless otherwise indicated

^a^Includes acute myocardial infarction, stable angina and atrial fibrillation

^b^Defined as an aspartate transaminase or alanine transaminase level of > 5× the upper normal limit or presence of hepatic failure

### Treatment discontinuation

Of the 105 patients who experienced AEs, 53.3% permanently discontinued treatment because of AEs (Table 4). Of all the patients who experienced AEs, 33.3% maintained treatment at the same dosage, 4.8% received a transient dose reduction, and 8.6% received a permanent dose reduction. The most frequent reasons for permanent discontinuation were disease progression (42.9%), anorexia (16.1%), and weight loss (10.7%; Table 5). The number of patients who permanently discontinued treatment as a result of AEs was significantly higher in the advanced group than in the non-advanced group (68.0% vs. 40.0%, *p* = 0.004; Table 4). However, with the exception of a high rate of anorexia in the advanced group (23.5% vs. 4.5%, *p* = 0.074; Table 5), no inter-group differences were found in the reasons for permanent discontinuation.

**Table 4:** Comparison of treatment interruption due to adverse events between the advanced and non-advanced idiopathic pulmonary fibrosis groups

Characteristic	Total	Advanced	Non-advanced	*P*-value
Number of patients	105	50	55	
Administration without dose reduction	35 (33.3)	10 (20.0)	25 (45.5)	0.006
AEs leading to transient dose reduction	5 (4.8)	0 (0.0)	5 (4.8)	0.058
AEs leading to permanent dose reduction	9 (8.6)	6 (12.0)	3 (5.5)	0.304
AEs leading to permanent discontinuation	56 (53.3)	34 (68.0)	22 (40.0)	0.004

Data are presented as number (%), unless otherwise indicated

*AEs* Adverse events

**Table 5:** Comparison of reasons for drug interruption due to adverse events between the advanced and non-advanced idiopathic pulmonary fibrosis groups

Characteristic	Total	Advanced	Non-advanced	*P*-value
Number of patients	56 (100.0)	34 (100.0)	22 (100.0)	
Disease progression^a^	24 (42.9)	16 (47.1)	8 (36.4)	0.430
Anorexia	9 (16.1)	8 (23.5)	1 (4.5)	0.074
Weight loss	6 (10.7)	2 (5.9)	4 (18.2)	0.198
General weakness	6 (10.7)	5 (14.7)	1 (4.5)	0.386
Diarrhoea	5 (8.9)	2 (5.9)	3 (13.6)	0.371
Nausea/vomiting	5 (8.9)	2 (5.9)	3 (13.6)	0.371
Pulmonary thromboembolism	3 (5.4)	1 (2.9)	2 (9.1)	0.555
Pneumonia	2 (3.6)	2 (5.9)	0 (0.0)	0.514

Data are presented as numbers (%), unless otherwise indicated

^a^Includes disease worsening, acute exacerbation and death

## Discussion

In the present study, nintedanib reduced the lung function decline in patients with IPF in a real-world setting. A comparable reduction in the decline of lung function was observed in the advanced and non-advanced groups. The majority of patients experienced AEs, and thus, half of these individuals were discontinued from treatment. In most cases, this was a result of disease progression. The frequency of AEs in the advanced group was similar to that in the non-advanced group. However, a higher frequency of treatment interruption was observed in the advanced group, mainly as a result of disease progression.

Data from phase 3 trials have indicated that nintedanib may be effective for the treatment of IPF, regardless of lung function [10–12]. However, these trials excluded patients with severely impaired lung function (FVC < 50% predicted or DLco < 30% predicted) [8] and the updated guidelines suggested that future trials should focus on this group of patients [1]. The present results showed that nintedanib delayed the decline in the lung function of patients with advanced IPF to an extent comparable with that observed in non-advanced cases. This is consistent with the results of previous studies [9, 18]. In a subgroup analysis of INPULSIS-ON, the absolute mean change in FVC from baseline to week 48 in patients (*n* = 24) with baseline FVC ≤ 50% predicted was similar to that in those (*n* = 558) with baseline FVC > 50% predicted (− 62.3 vs. -87.9 mL), which suggested that nintedanib may have a similar effect on disease progression in patients with advanced disease and those with less advanced disease [9]. A recent retrospective observational study, in 94 patients with IPF treated with nintedanib, also showed that median changes in FVC after 12 months were not different (∆ FVC: -6.52 vs. -2.60% predicted, *p* = 0.56) between patients with baseline FVC < 50% predicted (*n* = 9) and > 50% predicted (*n* = 48) [18]. However, the number of patients with advanced IPF was small [9, 18] and the efficacy analysis was descriptive [9]. The present study has therefore generated more robust evidence for the significant stabilising effect of nintedanib on the decline in lung function in patients with advanced IPF.

The present data indicated that nintedanib may be more effective for patients in the advanced group than for those in the non-advanced group. The rate of decline of FVC was significantly higher in the advanced group than in the non-advanced group before treatment, but this difference was not observed after treatment. A significant stabilisation of the FVC decline rate and a reduction in the rate of disease progression after treatment were also observed in the advanced group, but not in the non-advanced group. Previous reports supported our findings [10, 18]. In a pooled analysis of the INPULSIS trial, the results of the prespecified subgroup analyses (baseline FVC ≤ 70% predicted vs. > 70% predicted) suggested a more profound effect of the treatment in the advanced group for acute exacerbation (hazard ratio: 0.52 [≤ 70% predicted] vs. 1.00 [> 70% predicted], *p* = 0.175) and change from baseline in St. George’s Respiratory Questionnaire (difference in the adjusted changes between nintedanib and placebo: − 3.34 vs. -0.34, *p* = 0.063). Tzouvelekis et al. also demonstrated that the change in FVC after 12 months of treatment was significantly lower in patients with baseline FVC < 80% predicted than in those without (∆ FVC: 1.42% vs. -10.11% predicted, *p* = 0.03) [18]. In addition, previous studies suggested that pirfenidone, another antifibrotic agent used in the treatment of IPF, may be more effective in patients with a greater impairment of lung function, because they cause a greater decline in lung function before treatment [19–21]. Therefore, the absence of a significant reduction in the rate of decline of FVC or disease progression in the non-advanced group may be a result of the modest decrease in FVC before treatment; however, the rate of decline in TLC and the adjusted mean changes from baseline in FVC and TLC was significantly reduced after treatment in this group.

Although a significant reduction in the decline of FVC and TLC was observed in the present cohort, no such reduction was observed for DLco. The decline in DLco is an indicator of poor prognosis in IPF [2, 22]. However, DLco is also associated with pulmonary hypertension [22, 23]. Pulmonary hypertension is prevalent in patients with advanced IPF [24, 25]. Compared with the previous cohorts [8], patients in the present study had poor lung function (FVC: 79.5–80.0% vs. 56.2% predicted, DLco: 47–47.8% vs. 35.7% predicted), and one-third of the patients had suspected pulmonary hypertension (Table 1).

The incidence of AEs in the present cohort was similar to that reported in previous research (95.3% vs. 94.5–96.4%) [8]. Comparable rates were found for diarrhoea (61.5% vs. 61.5–63.2%), nausea (24.3% vs. 22.7–26.1%) and upper respiratory infection (9.0% vs. 9.1%). However, anorexia was more frequent in the present study (45.4% vs. 8.4–12.4%). Our findings reflect the real-world experience, and other studies of patients with IPF treated with nintedanib within a compassionate use programme have generated similar results (19.1–38.7%) [18, 26, 27]. Real-world studies [18, 26] usually include patients with more severely impaired lung function (FVC: 64–68% vs. 79.5–80.0% predicted) than phase 3 trials [8]. A previous study suggested that a high Gender-Age-Physiology score and a lower performance status were associated with the development of nausea or anorexia during the treatment of patients with IPF with nintedanib [28]. Concurrent medication may also contribute to the more frequent occurrence of anorexia in the real-world setting.

In the present study, the rate of diarrhea was higher in the non-advanced group than in the advanced group. This finding may be attributable to the higher exposure to nintedanib in the non-advanced group, which included more patients without dose modification and administered a longer duration of treatment. To minimize the difference in treatment duration between the two groups, we estimated adjusted ORs for AEs, and no significant difference in the risk of AEs was observed between the advanced and non-advanced groups. This finding was consistent with that of a previous report, which showed no significant difference in the rate of adverse reactions to nintedanib between advanced (FVC < 50% predicted or diffusing capacity < 30% predicted) and non-advanced groups [29].

Pneumonia was the most common serious adverse event in the present study, but the frequency was not different between the advanced and non-advanced groups. In the INPULSIS trials, the frequency of infection (56.3% [nintedanib] vs. 53.9% [placebo]) was also similar in both the nintedanib and placebo groups, which suggested that nintedanib was not associated with pneumonia [30]. In our study, the frequency of pneumonia was higher than in other study (27.8% vs. 3.1%) [31]. This result may be due to lower BMI of the patients in our study (BMI: 23.3 vs. 27.5 kg/m^2^) [31], and being underweight is generally associated with an increased risk of community acquired pneumonia (odds ratios 1.04–2.20) [32]. No inter-group difference was found in the risk of SAEs. However, the advanced group showed a tendency for a higher risk of pneumothorax (OR: 8.611; *p* = 0.068). In a recent study, pneumothorax in patients with IPF was associated with disease severity (presence of extensive reticular abnormalities) and poor outcome (hazard ratio, 2.85; *p* = 0.006) [33].

Compared with previous studies [8, 29, 34], the rate of discontinuation because of AEs in the present cohort was high (19–26.3% vs. 53.0%), which may be attributable to the severe disease status of a large proportion of the cohort. This was also consistent with the finding that disease progression was the most common cause of drug discontinuation. Diarrhea led to premature discontinuation in 4.6% of patients, which was consistent with findings from other phase 3 trials (4.3–4.5%). However, discontinuation owing to anorexia occurred more frequently in the present cohort (8.3% vs. 1.2–1.6%) [8]. The high discontinuation rate owing to anorexia in Asian patients in the INPULSIS trials (3.1% vs. 1.2–1.6%) [35] is an indication that racial or cultural differences may contribute to these findings. The number of patients that were permanently discontinued from the study owing to AEs was significantly higher in the advanced group than in the non-advanced group. This was consistent with a previous report, which found that a higher proportion of patients with FVC ≤ 50% predicted discontinued treatment owing to AEs (41.5% vs. 22.5%) and disease progression (17.1% vs. 5.4%) than patients with FVC > 50% predicted [9]. Low body weight is a factor that increases the exposure to nintedanib at equal doses [36]; therefore, the high treatment discontinuation rate in the advanced group may be related to this factor.

The present study has some limitations. First, the study was performed at a single center and included patients with varying disease severities and comorbidities. However, the demographic features and lung function of the cohort were comparable with those of other studies, and systematic history collection, physical examination and blood tests for possible AEs were performed on all patients at follow-up visits. Second, the number of patients was relatively small and patients in the advanced group were more frequently excluded from the efficacy analysis than those in the non-advanced group. Thus, there is a possibility of selection bias because the remaining patients might be better responders to treatment after the exclusion of patients who had difficulties performing pulmonary function tests or died. However, because the number of patients excluded was not large (*n* = 6), these effects would not have a significant impact on the overall results. Third, the study lacked a control group. However, a previous randomised placebo-controlled trial showed that nintedanib effectively reduced lung function decline in patients with non-advanced IPF [8].

## Conclusions

In conclusion, the present data suggest that nintedanib may stabilise lung function decline in patients with IPF treated in the real-world setting, irrespective of baseline lung function. The efficacy and safety profiles of nintedanib in the advanced group were comparable to those in the non-advanced group. Nintedanib could therefore represent a treatment option for patients with advanced IPF, especially those who are not candidates for lung transplantation. Further large-scale studies are warranted to confirm the present findings.

## Additional file

Additional file 1: **Table S1.** Inclusion and exclusion criteria of the named patient use programme for nintedanib. IPF, idiopathic pulmonary fibrosis; ATS, American Thoracic Society; ERS, European Respiratory Society; JRS, Japanese Respiratory Society; ALAT, Latin American Thoracic Association; AST, aspartate aminotransferase; ALT, alanine aminotransferase; INR, international normalised ratio. **Table S2.** Odds ratios for the development of adverse events, adjusted for treatment duration, in the advanced group compared with the non-advanced idiopathic pulmonary fibrosis group. OR, odds ratio; CI, confidence interval. * Compared with the non-advanced group. **Table S3.** Comparison of time-to-occurrence of adverse events between the advanced and non-advanced idiopathic pulmonary fibrosis groups. Data are presented as median [interquartile range, weeks], unless otherwise indicated. URI, upper respiratory infection. **Table S4.** Odds ratios for the development of serious adverse events, adjusted for treatment duration, in the advanced group compared with the non-advanced idiopathic pulmonary fibrosis group. OR, odds ratio; CI, confidence interval. * Compared with the non-advanced group. (DOCX 24 kb)

## Abbreviations

AEs: Adverse events
ATS: American Thoracic Society
BMI: Body mass index
CI: Confidence interval
DLco: Diffusing capacity of the lung for carbon monoxide
FGFs: Fibroblast growth factors
FVC: Forced vital capacity
IPF: Idiopathic pulmonary fibrosis
IQR: Interquartile range
OR: Odds ratio
PDGFs: Platelet-derived growth factors
SAEs: Serious AEs
SD: Standard deviation
TLC: Total lung capacity
