# Supplementary material for: Efficacy and safety of nintedanib in advanced idiopathic pulmonary fibrosis
Source: Respir Res. 2018 Oct 19;19:203. doi: 10.1186/s12931-018-0907-8 (PMC6194688; doi:10.1186/s12931-018-0907-8)
Supplement: Supplementary file 1 — Table S1. Inclusion and exclusion criteria of the named patient use programme for nintedanib. IPF, idiopathic pulmonary fibrosis; ATS, American Thoracic Society; ERS, European Respiratory Society; JRS, Japanese Respiratory Society; ALAT, Latin American Thoracic Association; AST, aspartate aminotransferase; ALT, alanine aminotransferase; INR, international normalised ratio. Table S2. Odds ratios for the development of adverse events, adjusted for treatment duration, in the advanced group compared with the non-advanced idiopathic pulmonary fibrosis group. OR, odds ratio; CI, confidence interval. * Compared with the non-advanced group. Table S3. Comparison of time-to-occurrence of adverse events between the advanced and non-advanced idiopathic pulmonary fibrosis groups. Data are presented as median [interquartile range, weeks], unless otherwise indicated. URI, upper respiratory infection. Table S4. Odds ratios for the development of serious adverse events, adjusted for treatment duration, in the advanced group compared with the non-advanced idiopathic pulmonary fibrosis group. OR, odds ratio; CI, confidence interval. * Compared with the non-advanced group. (DOCX 24 kb) [file 12931_2018_907_MOESM1_ESM.docx]

**Table S1. Inclusion and exclusion criteria of the named patient use programme for nintedanib**

| **Inclusion criteria** |
| --- |
| Age ≥ 40 years |
| IPF satisfying the ATS/ERS/JRS/ALAT diagnostic criteria for IPF (2011) |
| **Exclusion criteria** |
| AST, ALT > 1.5× upper limit of normal |
| Total bilirubin > 1.5× upper limit of normal |
| Conditions with increased bleeding risk |
| 1. Taking anticoagulation therapy |
| 2. INR > 2 |
| 3. Intracranial haemorrhage, stroke and transient cerebral infarction in the past 12 months |
| 4. Haemoptysis, haematuria, active gastrointestinal bleeding or ulcer in the past 3 months |
| 5. Congenital coagulopathy |
| 6. History of thrombosis in the past 12 months |
| Plan for lung transplantation or major abdominal surgery within the next 3 months |
| Heart disease |
| 1. Acute myocardial infarction in the past 6 months |
| 2. Unstable angina in the past 1 month |
| Concurrent use of investigational drugs |
| Concurrent use of high-dose prednisolone, other antifibrotic agents or immunosuppressants |
| Pregnancy or breastfeeding |

IPF, idiopathic pulmonary fibrosis; ATS, American Thoracic Society; ERS, European Respiratory Society; JRS, Japanese Respiratory Society; ALAT, Latin American Thoracic Association; AST, aspartate aminotransferase; ALT, alanine aminotransferase; INR, international normalised ratio.

**Table S2.** **Odds ratios for the development of adverse events, adjusted for treatment duration, in the advanced group compared with the non-advanced idiopathic pulmonary fibrosis group**

| Characteristic | OR* | 95% CI | p-value |
| --- | --- | --- | --- |
| Adverse events | 1.460 | 0.123–17.292 | 0.764 |
| Diarrhoea | 0.605 | 0.257–1.425 | 0.250 |
| Anorexia | 1.919 | 0.826–4.459 | 0.130 |
| Nausea/vomiting | 2.105 | 0.768–5.769 | 0.148 |
| Dyspepsia | 0.669 | 0.287–1.557 | 0.351 |
| Cough | 1.156 | 0.392–3.409 | 0.793 |
| Dyspnoea | 2.459 | 0.694–8.720 | 0.164 |
| Weight loss | 1.692 | 0.505–5.664 | 0.394 |
| General weakness | 1.843 | 0.516–6.578 | 0.346 |
| Upper respiratory infection | 0.728 | 0.182–2.903 | 0.652 |
| Hepatotoxicity | 2.400 | 0.568–10.142 | 0.234 |

OR, odds ratio; CI, confidence interval.

* Compared with the non-advanced group.

**Table S3. Comparison of time-to-occurrence of adverse events between the advanced and non-advanced idiopathic pulmonary fibrosis groups**

| Characteristic | Total | Advanced | Non-advanced | p-value |
| --- | --- | --- | --- | --- |
| Adverse events | 4.3 [1.6–11.7] | 3.9 [ 0.4–8.2] | 4.9 [3.0–15.0] | 0.373 |
| Diarrhoea | 13.9 [4.5–23.5] | 8.6 [2.9–20.1] | 15.0 [5.2–27.1] | 0.053 |
| Anorexia | 10.0 [4.3–33.9] | 5.0 [2.0–13.6] | 17.6 [7.2–43.0] | 0.042 |
| Nausea/vomiting | 4.1 [0.0–14.0] | 3.4 [0.0–14.0] | 4.3 [3.7–31.1] | 0.296 |
| Dyspepsia | 9.1 [3.7–25.6] | 5.5 [0.4–25.7] | 11.9 [5.6–22.4] | 0.749 |
| Cough | 16.7 [0.0–40.3] | 2.1 [0.0–11.5] | 30.0 [10.7–48.6] | 0.081 |
| Dyspnoea | 16.0 [6.2–20.6] | 10.6 [4.9–18.3] | 19.9 [18.4–21.8] | 0.303 |
| Weight loss | 23.0 [16.0–55.7] | 15.7 [10.1–32.9] | 37.7 [20.0–56.1] | 0.299 |
| General weakness | 10.0 [2.5–24.8] | 4.0 [0.0–16.3] | 16.0 [15.0–39.3] | 0.391 |
| URI | 27.3 [10.3–53.6] | 19.3 [8.5–31.5] | 52.0 [10.4–58.8] | 0.332 |
| Hepatotoxicity | 2.1 [0.9–128.1] | 3.2 [1.0–8.6] | 2.1 [0.9–128.1] | 0.450 |

Data are presented as median [interquartile range, weeks], unless otherwise indicated.

URI, upper respiratory infection.

Table S4. Odds ratios for the development of serious adverse events, adjusted for treatment duration, in the advanced group compared with the non-advanced idiopathic pulmonary fibrosis group

| Characteristic | OR* | 95% CI | p-value |
| --- | --- | --- | --- |
| Serious adverse events | 2.002 | 0.857–4.679 | 0.109 |
| Pneumonia | 1.277 | 0.508–3.209 | 0.603 |
| Pneumothorax | 8.611 | 0.851–87.135 | 0.068 |
| Pulmonary thromboembolism | 1.355 | 0.107–17.245 | 0.815 |
| Acute exacerbation | 1.322 | 0.310–5.716 | 0.700 |
| Ischaemic heart disease | 0.284 | 0.023–3.517 | 0.327 |
| Lung cancer | 1.909 | 0.133–27.334 | 0.634 |
| Death | 1.767 | 0.568–5.495 | 0.325 |

OR, odds ratio; CI, confidence interval.

* Compared with the non-advanced group.
